# Editorial: the role of mitochondrial endoplasmic reticulum contact sites in human health and disease

**DOI:** 10.3389/fmolb.2023.1223354

**Published:** 2023-05-23

**Authors:** Prasanna Katti, Sharifa Love-Rutledge, Sandra A. Murray, Antentor Hinton

**Affiliations:** ^1^ National Heart, Lung and Blood Institute, National Institutes of Health, Bethesda, MD, United States; ^2^ Department of Chemistry, University of Alabama in Huntsville, Huntsville, AL, United States; ^3^ Department of Cell Biology, School of Medicine, University of Pittsburgh, Pittsburgh, PA, United States; ^4^ Department of Molecular Physiology and Biophysics, Vanderbilt University, Nashville, TN, United States

**Keywords:** mitochondria, MERCs, disease, contact sites, pathology

## Introduction

This editorial aims to consider mitochondrial factors in Endoplasmic Reticulum (ER) stress with a comprehensive, organ-based overview of human biology and disease pathophysiology from a clinical perspective. Intracellular interactions form an intricate network, with inter-organellar communication pivotal in regulating cellular physiology. A key focus of recent research has been the crosstalk between two essential organelles, mitochondria, and the ER, facilitated through specialized contact sites known as Mitochondria-endoplasmic reticulum contacts (MERCs). MERCs maintain cellular homeostasis and orchestrate various cellular processes, making it vital to understand their complexities and dynamics within the broader context of cell biology. Beyond only considering the role of these MERCs, this editorial considers how MERCs are involved in insulin-mediated structural changes, metabolism, and other biochemical roles. Specifically, by taking a novel approach, this editorial highlights manuscripts that address developing technologies to study these contact sites including transmission electron microscopy, 3D electron microscopy, super resolution techniques to identify and quantify organelle structure. Finally, this editorial also considers novel regulators of MERCs in TMEM 135 and the MICOS family of genes and how these may function in tissue-specific age-related structural changes.

**FIGURE 1:**
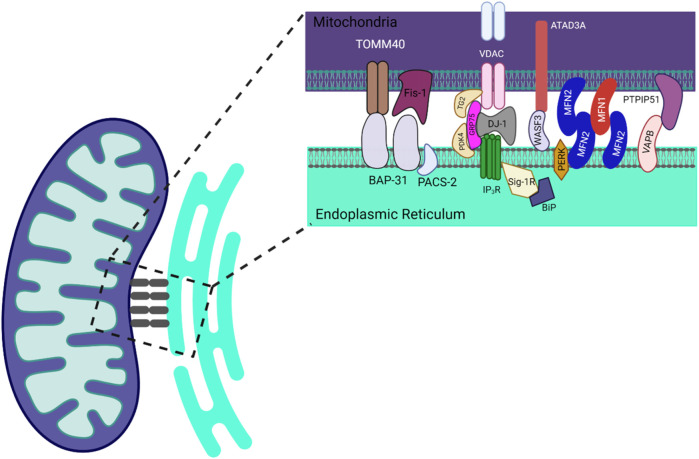
Schematic illustration of the distribution of proteins at the mitochondrial-endoplasmic reticulum (ER) contact sites. Figure Created with Biorender.

Shore and Tata introduced the concept of physical connections between the ER and mitochondria over 4 decades ago through their electron microscopy studies on liver mitochondria (Shore and Tata, 1977). Later, Csordás et al. utilized electron tomography to reveal diverse “tethers” connecting the outer mitochondrial membrane to various regions of the smooth and rough ER (Csordas et al., 2006; Csordas et al., 2018). Identifying the protein complexes that constitute MERCs has greatly advanced our understanding of the molecular mechanisms underpinning MERC formation and their myriad roles in cellular physiology. MERCs comprise an assortment of proteins, including MFN2 (mitofusin 2), IP3R3 (inositol 1,4,5-trisphosphate receptor type 3), GRP75 (glucose-regulated protein 75), VAPC (vesicle-associated membrane protein-associated protein C), and others (Csordas et al., 2006; Csordas et al., 2018; Glancy et al., 2020). These proteins facilitate vital interactions between mitochondria and the ER by forming multiprotein complexes such as IP3R1-GRP75-VDAC1, MFN1-MFN2, and Fis-Bap31 complexes (Figure 1) (Lopez-Crisosto et al., 2017). MERCs enable the exchange of lipids, calcium, and other molecules essential for maintaining organelle functions, playing pivotal roles in calcium homeostasis, lipid metabolism, and autophagy regulation. This editorial article explores the interdependent worlds of mitochondria and the ER, emphasizing the significance of MERCs. Despite their importance, our understanding of MERCs is still limited, necessitating further research into their roles in disease and their potential as therapeutic targets.

Our Special Research Topic aims to offer a comprehensive perspective on human biology and disease pathophysiology from a clinical standpoint, focusing on diseases associated with mitochondrial dysfunction. This editorial introduces our Research Topic, emphasizing the impacts of mitochondrial dysfunction and MERCs on health conditions such as insulin resistance, skeletal muscle fat accumulation, and age-related organ function changes. Our goal is to facilitate the development of innovative therapeutic strategies by thoroughly examining mitochondrial-ER contact sites and their implications for health and disease. Noteworthy contributions include Nieblas et al. Highlighted MAM’s role in insulin signaling and metabolic regulation. Assis et al. showed hypercholesterolemia and pravastatin’s impact on macrophages’ mitochondrial-ER interaction. Gansemer and Rutkowski reviewed how ER function connects to metabolic activity through oxidative protein folding and NADPH generation. Saneto and Perez illustrated how MAM structures’ genetic variants, including mutations in MICU1, PASC-2, CYP2U1, SERAC1, and TANGO2, can cause diseases and affect CNS structures. Morgado-Cáceres et al. examined MERCs’ progression from undifferentiation to specialization and their relevance to chronic age-related diseases. Hogan et al. studied organelle dysfunction in age-related diseases, focusing on mitochondrial Research Topic and metabolic flux alterations. Lastly, Wang et al. demonstrated that disrupting the GRP75 protein, linking ER and mitochondria, impairs adipogenic differentiation and increases ROS in 3T3-L1 preadipocytes.

## Highlights

### The role of impaired mitochondrial dynamics in MFN2-Mediated pathology

Zaman and Shutt explore the implications of MFN2 dysfunction in Charcot-Marie-Tooth disease type 2A (CMT2A), a peripheral neuropathy. Despite MFN2’s multiple roles, only a handful of the 100+ pathogenic variants associated with CMT2A have been studied functionally. These mutations do not uniformly affect MFN2 functions. Impaired MERCs and reduced mitochondrial mobility are key features of these variants, contributing to the CMT2A pathology. Disrupted MERCs and abnormal mitochondrial distribution are tied to impaired Ca2+ homeostasis and decreased axonal mitochondrial movement. However, the diverse roles of MFN2, such as lipid metabolism and mitophagy, necessitate further research to understand its dysfunction in CMT2A.

### Disruption of mitochondria-associated ER membranes impairs insulin sensitivity and thermogenic function of adipocytes

Wang and colleagues' study highlights the crucial role of MAMs in adipocytes’ function and metabolism. By disrupting MAM structure in 3T3-L1 adipocytes, the team found impaired differentiation, functionality, and insulin sensitivity, along with overproduction of reactive oxygen species (ROS). This disruption also triggered a cycle of ROS accumulation, suggesting potential therapeutic benefits from antioxidant treatments. The research further revealed that brown adipocytes, possessing a higher capacity for glucose/fatty acid uptake, utilization, and insulin response than their white counterparts, heavily rely on MAMs. Disruption of MAMs in these cells impaired their thermogenic function and resulted in obesity and glucose dysregulated homeostasis in mice. Some MAM proteins, including PERK and Seipin, were found to be key players in these processes. This study underscores the importance of MAMs in cellular metabolism and highlights their potential as therapeutic targets for metabolic disorders like obesity and Type 2 diabetes. Despite these advances, further research is needed to fully understand the mechanisms linking MAM formation and brown adipocyte function.

### Aging and ER-mitochondria communication

Morgado-Cáceres et al. illuminate the diverse functions of MERCs across various cell types. Integral to cellular processes, including mitochondrial distribution, calcium regulation, and lipid metabolism, MERCs bridge the ER and mitochondrial networks. Their role in disease progression offers promising therapeutic potential for age-related conditions. MERCs display dynamic structural and functional diversity throughout a cell’s lifecycle. They ensure proper mitochondrial distribution in proliferating cells and regulate intracellular Ca2+ signals in differentiating ones. Their role in cancer cell viability is complex and warrants further investigation.

In differentiated cells, MERCs contribute to functions like fatty acid uptake, angiogenesis, ATP provision, immune response activation, and lipid metabolism. Understanding MERCs’ role in disease progression is imperative given their alteration in numerous pathologies. The review accentuates the therapeutic potential of MERCs in age-related diseases. However, due to their complexity, further research is needed to develop safe, efficient, cell-specific MERC-targeted therapeutic approaches. This understanding could catalyze new strategies to combat metabolic and age-related disorders, potentially improving millions of lives worldwide.

### Hypercholesterolemia and MAM contact sites in macrophages

The study by Assis et al., examines the intricate relationship between hypercholesterolemia, pravastatin treatment, and MAM within bone marrow-derived and peritoneal macrophages. Hypercholesterolemia amplifies the number of ER-mitochondria contact sites, potentially triggering calcium influx towards mitochondria, while pravastatin treatment restores contact site number and hinders the induction of trained immunity memory in bone marrow-derived macrophages. The research scrutinizes hypercholesterolemia and pravastatin’s effects on oxidant production, mitochondrial respiration rates, and inflammation-linked gene expression. Unexpectedly, pravastatin enhances superoxide anion production without affecting hydrogen peroxide release, suggesting it may elevate oxidative stress. It also heightens pro-inflammatory cytokine IL-1β expression, potentially causing damage to other cells.

The study also investigates how hypercholesterolemia and pravastatin influence the IP3R1-VDAC1 complex, a key tethering entity in ER-mitochondria interaction. However, the study does not fully elucidate whether changes extend to overall MAM stability or are solely confined to the IP3R1-VDAC1 complex. Furthermore, the use of a chronically hypercholesterolemic mouse model raises questions about pravastatin’s mechanism of action on MAM stability. This study underscores the need for continued research to discern precise mechanisms and potential therapeutic implications for hypercholesterolemia and atherosclerosis treatment. It also advocates for careful patient monitoring when administering pravastatin to ensure its safety and efficacy.

### NADPH, ER protein oxidation, and stress

This extensive review by Gansemer & Rutkowski sheds light on the complex pathways of NADPH production and their interaction with oxidative protein folding and metabolism. It highlights ER functions, such as disulfide bond formation, signal peptide cleavage, and N-linked glycosylation, which are sensitive to NADPH status changes. Deciphering the chief pathway linking NADPH and ER function is challenging due to the numerous redox pathways NADPH controls. Future studies must identify how these pathways individually or collectively affect ER oxidation capacity and clarify oxidized glutathione’s role in ER protein oxidation.

The review emphasizes the need to explore the relationship between metabolic fluxes and redox-active metabolites, which could uncover links between these processes. It also discusses the role of redox signals across membranes, particularly IDH1 and IDH2, and how the cellular redox state affects ER client proteins. Understanding why the unfolded protein response reacts to catabolism-produced metabolites and whether the ER operates better under more oxidizing or reducing conditions is essential. Furthermore, the review discusses the ER’s role in calcium storage and regulated release, stressing the importance of Sarco/Endoplasmic calcium ATPase proteins, which are redox-sensitive. However, the molecular mechanisms linking metabolism to SERCA activity and their connection to ER redox pathways require further clarification. The review hypothesizes that increased nutrient catabolism leads to NADPH production, causing a hypo-oxidizing ER, primarily through glutathione and possibly thioredoxin pathways.

Notably, this review suggests that a deeper understanding of the intricate interplay between NADPH, nutrient availability, and ER protein processing could lead to novel treatments for ER stress and related diseases. Examining the roles of thioredoxin reductases in cellular processes could lead to therapies for diseases associated with NADPH oxidases, which produce reactive oxygen species, contributing to conditions such as cardiovascular disease, inflammation, and cancer.

### Mitochondria-associated membrane scaffolding with endoplasmic reticulum

As Saneto and Perez detail, MAMs are involved in various cellular processes, including MICU1-mediated calcium homeostasis, PASC-2-assisted lipid transfer, SERAC1-regulated mitochondrial dynamics, CYP2U1-driven mitophagy and apoptosis, and TANGO2-involved Golgi-ER communication.

The authors present case studies involving children with genetic variants in MAM-related proteins demonstrate the severe diseases that MAM dysfunction can induce, characterized by neurocognitive delay, developmental Research Topic, muscular problems, and neuroimaging abnormalities. These cases underscore MAMs’ essential role in neurodevelopment and the diverse disease phenotypes linked to MAM disruptions. One study highlighted calcium regulation’s importance in two brothers with global developmental delay, hypotonia, and elevated enzyme levels. Another case report associated a PACS-2 gene variant with cellular homeostasis disruption. A case involving SERAC1, crucial for lipid remodeling and cholesterol trafficking, related to a child with MEGDEL syndrome symptoms. CYP2U1 variants, cytochrome P450 proteins, were tied to hereditary spastic paraplegia (SPG-56), indicating MAM dysfunction’s effect on disease progression. TANGO2 deficiency showcased wide-ranging symptoms, reflecting the condition’s clinical variability. In summary, these cases emphasize MAMs’ critical role in cellular health, the implications of their dysfunction on disease progression, and the potential of MAMs as therapeutic targets. The diversity in clinical presentations and disease progressions underscores a broader loss of interconnected MAM functioning. Thus, the evolving understanding of MAMs in physiology and disease presents opportunities for developing targeted interventions for these complex disorders.

### Mitochondria-associated endoplasmic reticulum membranes in insulin sensitivity, energy metabolism, and contraction of skeletal muscle

Nieblas et al. comprehensively review the critical role of MAMs and MERCs in skeletal muscle contraction and metabolism. They highlight the significance of mitochondrial dysfunction and ER stress in the genesis of insulin resistance (IR) in skeletal muscle. Mitochondria, the primary energy producers, and the ER, accountable for protein and lipid synthesis and calcium homeostasis, are structurally and functionally interconnected *via* MAMs, contact sites comprising resident and cytosolic proteins.

Three distinct mitochondrial populations in muscle cells include subsarcolemmal mitochondria (SSM), intermyofibrillar mitochondria (IMF), and perinuclear mitochondria (PNM). Each has unique protein compositions, lipids, and oxidative capacities. IMF specializes in energy production for contractile function, while SSM is involved in fatty acid oxidation, glucose transport, and insulin signaling. MAMs provide a communication platform between the ER and mitochondria, especially for Ca^2+^-mediated signaling, lipid biosynthesis, energy control, and mitochondrial quality control. They play a crucial role in insulin signaling and insulin sensitivity in skeletal muscle. MAM integrity disruption can lead to insulin action loss, affecting muscle response to nutrient availability, and inducing insulin resistance. Exercise improves insulin sensitivity, mitochondrial density, and type I-to-type II fiber ratio, whereas obesity, metabolic syndrome, and type 2 diabetes decrease this ratio, reducing insulin sensitivity.

Oxidative insults trigger cellular stress responses, with mitochondria acting as early indicators. Mitochondrial dysfunction is now seen as altered inter-organelle communication in insulin-resistant tissues. Further study into inter-organelle communication concerning mitochondrial dysfunction and IR is crucial. This emerging field suggests modulating organelle interactions as a promising strategy for treating metabolic diseases. MAMs could be therapeutic targets for interventions like diet and physical activity, providing new approaches for treating metabolic disorders. As our understanding of organelle interplay in IR conditions grows, so does the potential for innovative therapies addressing the root causes of these diseases.

### Mass spectrometry imaging to visualize age-related subcellular disruption

Hogan et al. describes the use of mass spectrometry imaging (MSI) to investigate age-related subcellular disruptions, including links between mitochondrial metabolism and senescence. Disruptions in the tricarboxylic acid (TCA) cycle and NAD + metabolism contribute to mitochondrial decay in aging cells, while oxidative damage and reduced Ca^2+^ buffering affect ER functions, leading to deposits associated with age-related diseases. MSI’s techniques allow visualization of cellular and subcellular metabolism in disease models, which is beneficial for studying immunometabolism, cancer, and microbial resistance. In conclusion, the authors suggest that MSI revolutionizes our understanding of aging and age-related diseases, providing high-resolution spatial information on various biomolecules within cells and tissues.

### Closing comments

This Research Topic offers a profound analysis of MERCs, deepening our understanding of their role in cellular physiology and disease pathophysiology. The manuscripts in this Research Topic highlighted disease-related changes in MERC structure and the necessity of future organ-based investigation of disease pathophysiology from a clinical perspective. Importantly, there is an understudied regulators of mitochondrial function such as gap junction related to organelle contacts. Through investigating these mechanisms with new technologies, the roles of MERC dysfunction in Type 2 Diabetes, obesity, cardiovascular disease, and aging can be better understood. These insights may guide potential therapeutic strategies to improve health and address diseases related to mitochondrial-ER dysfunction.
